# Pheochromocytoma Presenting as Partial HELLP Syndrome

**DOI:** 10.1155/2015/294326

**Published:** 2015-08-16

**Authors:** Yazan Daaboul, Serge Korjian, Lamis Khalil, Rita Nemr

**Affiliations:** Lebanese American University Medical Center-Rizk Hospital, Gilbert & Rose-Marie Chagoury School of Medicine, Lebanese American University, Byblos, Lebanon

## Abstract

Diagnosis of pheochromocytoma in partial HELLP syndrome is extremely rare. We report a case of a 25-year-old multigravida woman at 30 weeks of gestation who presented with clinical features consistent with partial HELLP syndrome. Her symptoms were not controlled by pharmacologic therapy, and the patient underwent urgent cesarean section. The patient gave birth to a viable baby, but she sustained an episode of ventricular fibrillation intraoperatively that did not result in any long-term sequelae. The patient's symptoms persisted postoperatively and work-up for secondary etiologies of hypertension demonstrated a right adrenal pheochromocytoma. Following resection, the patient's signs and symptoms resolved, and her lab tests normalized.

## 1. Introduction

Pheochromocytoma is a rare catecholamine-producing tumor that afflicts 0.1 to 0.6% of patients who present with hypertension [1]. Diagnosis of pheochromocytoma in pregnancy is extremely rare with an approximate incidence of less than 0.01% [2]. It is often missed owing to the more commonly encountered forms of new-onset hypertension during pregnancy, namely, gestational hypertension and preeclampsia [3]. We report the case of young woman at 30 weeks of gestation with undiagnosed pheochromocytoma who presented with signs and symptoms consistent with partial HELLP syndrome (hemolysis, elevated liver enzymes, and low platelets).

## 2. Case Presentation

A 25-year-old pregnant woman, gravida 4-para 2-aborta 1, at 30 weeks of gestation, was referred to our center for headache and vomiting with elevated liver enzymes. Her past medical history was significant for episodic palpitations and tremor that started shortly following her last delivery two years prior to presentation. In the emergency department, she was found to have an elevated blood pressure of 230/110 mm Hg. Initial work-up demonstrated elevated liver transaminases (AST = 82 U/L; ALT = 230 U/L) and elevated total bilirubin = 2.5 mg/dL with a normal platelet count and serum creatinine concentration. Proteinuria was evident on urine dipstick analysis. Abdominal ultrasound was remarkable for a well-circumscribed perihepatic mass suggestive of hemangioma measuring 7.7 × 4.8 cm, with no gestational abnormalities. Given the clinical findings, the patient was admitted for suspected HELLP syndrome. On admission, the patient was fluid-restricted to 1.5 L of normal saline per day to prevent potential fluid overload. A bolus and a maintenance infusion of magnesium sulphate were administered for seizure prophylaxis, and the patient was continuously monitored for signs and symptoms of magnesium toxicity. Additionally, her elevated blood pressure was managed by repeated oral doses of nifedipine 10 mg, methyldopa 2 g daily, and continuous infusion of nitroglycerin. The following day, the patient was still symptomatic with uncontrollably elevated blood pressure despite optimization of her antihypertensive therapy. Her hemoglobin concentration dropped and her liver function tests increased further, but her platelet count remained within normal limit. Twenty-four-hour urine collection demonstrated marked proteinuria of 400 mg/dL and confirmed the diagnosis of partial HELLP (hemolysis, elevated liver enzymes, and low platelets) syndrome.

The patient underwent urgent cesarean section, and general anesthesia was provided due to urgency and inadequacy of regional block. Following successful delivery of a viable baby, the patient's intraoperative course was complicated by an episode of ventricular fibrillation, during which the patient was successfully resuscitated. Postpartum, the patient's symptoms did not improve, and work-up for secondary hypertension was performed. Elevated urinary concentrations of vanillylmandelic acid, metanephrine, and normetanephrine with the presence of a well-circumscribed right adrenal mass measuring 7.5 × 5.6 × 4.2 cm on abdominal MRI confirmed the diagnosis of pheochromocytoma. The patient was stabilized with alpha-blockade followed by beta-blockade, and adrenalectomy was then performed 15 days postpartum. At discharge, the patient's signs and symptoms resolved, and her lab tests normalized.

## 3. Discussion

Missed pheochromocytoma carries a high risk of mortality for both the mother and the fetus. Timely diagnosis and management of pheochromocytoma during pregnancy are crucial for the prevention of maternal and neonatal morbidity and mortality. Pheochromocytoma crises account for significantly high rates of maternal mortality that may range from 5% to 50%. Beyond maternal complications, fetal demise has been reported among approximately 15% of cases [4, 5].

In this patient, the initial diagnosis was partial HELLP syndrome. Sibai [6] suggested criteria for the diagnosis of HELLP syndrome that included the combination of hemolysis associated with microangiopathic hemolytic anemia (either abnormal peripheral blood smear with evidence of hemolysis, elevated total bilirubin > 1.2 mg/dL, or lactate dehydrogenase (LDH) > 600 U/L), elevated liver function tests (either AST > 70 U/L or LDH > 600 U/L), and low platelet count < 100,000/mm^3^. Accordingly, partial HELLP syndrome is diagnosed when not all the criteria for HELLP syndrome are met.

Although pheochromocytoma during pregnancy is often distinguished from preeclampsia and HELLP syndrome by its early and paroxysmal presentation during pregnancy prior to 20 weeks of gestation, the patient's normal follow-ups during her routine obstetric visits made the diagnosis of pheochromocytoma more difficult [4]. Furthermore, the presence of clinical findings consistent with partial HELLP syndrome (drop in hemoglobin, elevated liver transaminases, and significant proteinuria and normal platelet count) made the diagnosis of partial HELLP syndrome more likely. Nonetheless, the persistence of symptoms postpartum was not typical of partial HELLP syndrome and suggested that further work-up for alternative etiologies of hypertension was necessary.

Remarkably, neither the patient nor the fetus in this case sustained long-term adverse outcomes, given that both a postnatal diagnosis of pheochromocytoma and an intraoperative pheochromocytoma crisis may significantly increase the risk of maternal and neonatal morbidity and mortality [7–10]. Although the patient sustained an episode of ventricular fibrillation during cesarean section, she did not suffer any long-term sequelae. It is unknown what might have triggered the patient's arrhythmia during her delivery, but it is presumed that both the surgery and the administration of general anesthetic agents may have potentially triggered a pheochromocytoma crisis with significant hemodynamic disturbances [8, 11].

To our knowledge, this is the first report of pheochromocytoma in pregnancy with a presentation of partial HELLP syndrome. It is unclear whether a partial HELLP syndrome and a pheochromocytoma crisis overlap occurred or whether an acute catecholamine surge was responsible for the patient's full clinical syndrome. Pheochromocytoma is not typically associated with elevated liver enzymes, anemia, and proteinuria; however, solid data is lacking to clearly delineate the relationship between both diseases.

## 4. Conclusion

Pheochromocytoma and HELLP syndrome may coexist and manifest late in pregnancy with similar symptoms. Subsequently, obstetricians should have a low threshold of suspicion to work up pregnant women with uncontrolled hypertension and palpitations for pheochromocytoma especially if they report paroxysmal symptoms that may be missed on regular exam.
